# Return to work outcomes in solid organ transplant recipients: A protocol for a global scoping review

**DOI:** 10.1371/journal.pone.0319873

**Published:** 2026-04-08

**Authors:** Vinícius Araújo Pereira, William Donegá Martinez, Martins Fideles dos Santos-Neto, Thaysa Castro Molina, William José Duca, Paulo César Arroyo-Júnior, Allana C. Fortunato, Adília Maria Pires Sciarra, Andressa Karina Stefani, Alex Bertolazzo Quitério, Sônia Maria Maciel Lopes, Luiz Otávio Maciel Lopes, Lucian Borges Lázaro, Flávia Cristina Custódio, Isabela Amaral de Almeida Bistafa, José Nathan Fernandes Rocha, João Daniel De Souza Menezes, Matheus Querino da Silva, Emerson Roberto dos Santos, Licia Paula Schelbauer Borges, Gabriela Gouvea Silva, Vania Del Arco Paschoal, Maysa Alahmar Bianchin, Luís Cesar Fava Spessoto, Heloísa Cristina Caldas, Fernando Nestor Facio Júnior, Júlio César André, Renato Ferreira da Silva

**Affiliations:** 1 Faculty of Medicine of São José do Rio Preto, São José do Rio Preto, Brazil; 2 Cancer Hospital of Barretos, Barretos, Brazil; 3 São José do Rio Preto Regional Medical School Foundation, São José do Rio Preto, Brazil; 4 Holy House of Mercy of Votuporanga, Votuporanga, Brazil; Public Library of Science, UNITED KINGDOM OF GREAT BRITAIN AND NORTHERN IRELAND

## Abstract

Solid organ transplantation treats end-stage organ failure, improving longevity and quality of life. Return to work post-transplant is a positive indicator of treatment success. However, labor is influenced by multiple biopsychosocial factors, leading to complex barriers that affect recipients’ opportunities. Mapping return-to-work literature may reveal gaps in conceptualization, instruments, analyses, and key determinants. Following Joanna Briggs Institute methodology, this protocol aims to identify knowledge gaps and map the processes and outcomes in the literature on return to work after liver, kidney, heart, and lung transplantation. Following the Population, Concept, and Context strategy, this review is guided by the research question: “What has the literature shown about return to work after solid organ transplantation?”. This protocol was created and recorded on the Open Science Framework under DOI 10.17605/OSF.IO/Q6HVT. Database selection and search strategy were determined by a librarian specializing in health sciences. The literature search will be conducted in PubMed, Scopus, EMBASE, LILACS, and Web of Science databases. Eligible studies include primary and secondary research on return to work after transplantation, published in English or Portuguese, with no time restrictions. Two reviewers will independently perform the selection and data extraction. The data will be extracted using a pre-designed form to collect key details about the studies’ origin, context, purpose, content, population, and variables related to the return-to-work process. These data will be synthesized following Synthesis Without Meta Analysis guidelines and summarized narratively using tables, graphs, alongside thematic analysis.

## Introduction

Solid organ transplantation has emerged as the central treatment for organ failure and end-stage diseases involving the liver, kidney, heart, and lung. Its objective is to improve longevity and quality of life while reducing the impact of diseases and the costs associated with care [1]. In this context, return to work has increasingly been recognized as a key long-term functional indicator of treatment success after solid organ transplantation, with positive financial, psychosocial, and clinical implications [2–6]. Furthermore, it endorses the importance of investments in vocational rehabilitation programs and the economic sustainability of transplant services [7].

Beyond clinical factors, the possibility of labor reintegration and the development and sustainability of work rehabilitation within transplant programs are shaped by structural contextual conditions. These include economic (income and social inequality), geographic (municipal, regional, and national), and political factors (orientation of public policies toward social welfare and public health, among others), as well as legal and institutional differences in access to social determinants of health [2,3,8]. This characterizes challenges and barriers whereby labor return is far from uniform among transplant recipients [1,4].

The assessment of return to work after transplantation remains methodologically heterogeneous, which limits cross-study comparability and the identification of key determinants [7]. This is especially relevant as the literature indicates the influence of variables with biopsychosocial scope, such as the effect of immunosuppressants, pre-transplant diagnosis, post-transplant complications, depression, anxiety [1,4,6,7], social security system, access to benefits, age, sex, education, physical effort in professional activities, pre-transplant unemployment, inaccessible labor market, among others [2–4,6,9,10].

A preliminary search in the National Library of Medicine (PubMed) and Scopus (Elsevier) databases, conducted in May 2024, did not identify recent scoping reviews specifically addressing return to work after multiple solid organ transplants. In addition, there is a limited number of recent reviews on this topic, and the available studies lack critical analyses or consideration of possible biases in the scope of return to work [2,11].

Furthermore, there appears to be a current lack of standardized and psychometrically validated instruments to assess this phenomenon, which may contribute to variability in core concepts such as work and unemployment across studies on return to work. This scoping review will address this gap by mapping how employment, unemployment, and return to work have been defined and measured across studies involving different transplanted organs, supporting the development of more coherent and homogeneous conceptual frameworks and informing subsequent psychometric validation efforts. Additionally, the global scope of the synthesis may help identify cross-national differences in return-to-work patterns, including disparities related to legal frameworks and social protection systems, thereby informing clinical follow-up, rehabilitation planning, service organization, and the design of transplant-specific public policies aimed at labor reintegration.

Thus, this scoping review aims to map the existing literature, identify knowledge gaps, and explore key concepts related to return to work post-transplantation, providing a foundation for future research, new methods, psychometric instruments, and policies for this crucial area.

## Materials and methods

### Type of study

This is a scoping review that will be conducted according to the methodological frameworks proposed by the Joanna Briggs Institute (JBI) for scoping reviews [12]. The review process will adhere to the following steps: 1 – formulation of the research question; 2 – identification of relevant studies; 3 – study selection; 4 – data extraction; 5 – synthesis and reporting of results; and 6 – expert consultation.

### Protocol registration

This protocol was created and recorded on the Open Science Framework (OSF) [13] under DOI 10.17605/OSF.IO/Q6HVT.

### Ethics statement

This study is a scoping review protocol that does not involve direct human participant’s research. It utilizes only publicly available, previously published information. Therefore, institutional review board approval was not required or sought for this study.

### Research question

What has the literature shown about return-to-work after solid organ transplantation?

### Objective

To identify knowledge gaps and map biopsychosocial processes and outcomes in the literature on return to work after liver, kidney, heart, and lung transplantation, considering heterogeneity across transplant types, transnational contexts, and diverse methodological approaches.

### Inclusion criteria

Studies that centrally analyze the return-to-work process after solid organ transplantation will be included, encompassing primary research such as observational and experimental studies, as well as secondary studies like systematic reviews and meta-analyses. Both qualitative, quantitative, and mixed-methods approaches will be considered. No hierarchy will be established among the types of studies, with all being equally considered for the literature mapping. Eligible studies must be published in English or Portuguese, consistent with the researchers’ language proficiency, with no restrictions on publication date or country of origin.

### Exclusion criteria

Studies in which return to work is not explicitly defined as a primary outcome or research objective will be excluded. Gray literature, including theses and dissertations, as well as duplicate publications, letters to editors, abstracts, and opinion articles, will not be considered. Pediatric and cell transplant studies and any research not specifically related to liver, kidney, heart, or lung transplantation will also be excluded.

### Eligibility criteria

#### Research strategy.

Widely applied in scoping and systematic reviews, the Population, Concept, and Context strategy was used to formulate the research question [14].

#### Population.

The population of interest for this scoping review includes adult (≥18 years) **recipients of solid organ transplants**, specifically liver, kidney, heart, and lung. Studies with participants of all sexes and gender identities will be considered, regardless of pre-transplant employment status. The review will cover studies evaluating return to work at any post-transplant period, from the immediate postoperative phase to the long-term (>5 years). Both participants who were employed prior to transplantation and those seeking employment after the procedure will be included.

#### Concept.

The central concept examined in this scoping review is the **return to work after solid organ transplantation**. For the purposes of this study, ‘return to work’ is defined as the resumption of any form of paid employment, whether full-time, part-time, or self-employment, after transplantation. The concept encompasses not only the act of returning to work but also related aspects such as employability, workability, vocational rehabilitation, and job retention post-transplantation.

#### Context.

The context of this scoping review is broad and global. **Studies from all geographic regions** will be considered without restrictions, providing a worldwide perspective on the return to work after solid organ transplantation. The review will include research conducted in various settings, such as transplant centers, post-transplant follow-up clinics, workplaces, and communities. Additionally, the review will encompass studies from countries with diverse healthcare systems and transplant policies, enabling comparisons between different models of care and support.

### Databases and search strategy

The search strategies were developed in collaboration with a librarian specializing in health sciences. To ensure precision, accuracy, and comprehensiveness in the research, the controlled vocabulary MeSH was used for the PubMed database and the controlled vocabulary EMTREE for the Excerpta Medica Database (EMBASE). The searches will be conducted in November 2025. The strategies may be refined during the review process to optimize the sensitivity and specificity of the search.

The search strategy followed the three steps recommended by JBI:

1. **Initial limited search:** An initial search was conducted in the PubMed and Scopus databases to identify relevant articles on return to work after solid organ transplantation. The indexing terms and keywords used in these articles were analyzed to refine the search strategy.
2. **Expanded second search:** Based on the results of the initial search, a comprehensive search will be carried out across all included databases, using all identified indexing terms and keywords.
3. **Reference list search:** The reference lists of the selected articles will be examined to identify additional relevant studies that may not have been captured through electronic search.

The descriptors used in the search were “Employment,” “Return to Work,” and “Transplantation.” The databases consulted included PubMed, Scopus, EMBASE, Literatura Latino Americana e do Caribe em Ciências da Saúde (LILACS), and Web of Science, ensuring comprehensive coverage of the literature relevant to the research. The initial search strategy used these descriptors associated with the Boolean operators AND, OR, and NOT, being customized for each database.

The detailed approach to the search strategy is exemplified in Table 1, which outlines the methodology tailored for the PubMed database. Furthermore, this strategy, along with those applied to other databases is available in S1 Table. Search Strategy.

**Table 1:** Search strategy in PubMed.

Search strategy used for PubMed
(“Return to Work”[Title/Abstract] OR “Work, Return to”[Title/Abstract] OR “Back-to- Work”[Title/Abstract] OR “Return-to-Work”[Title/Abstract] OR “Back to Work”[Title/Abstract] OR “Work, Back to”[Title/Abstract] OR Employment[Title/Abstract] OR “Employment Termination”[Title/Abstract] OR “Termination, Employment”[Title/Abstract] OR “Labor Force”[Title/Abstract] OR “Labor Forces”[Title/Abstract] OR “Precarious Employment”[Title/Abstract] OR “Employment, Precarious”[Title/Abstract] OR “Marginal Employment”[Title/Abstract] OR “Employment, Marginal”[Title/Abstract] OR “Employment Insecurity”[Title/Abstract] OR “Employment Insecurities”[Title/Abstract] OR “Insecurity, Employment”[Title/Abstract] OR “Employment Status”[Title/Abstract] OR “Status, Employment”[Title/Abstract] OR “Status, Occupational”[Title/Abstract] OR ”Occupational Status”[Title/Abstract] OR Underemployment[Title/Abstract]) AND Transplantations NOT (Cell OR Cells)

Source: Authors, 2024.

### Study selection

The study selection process will be conducted independently by two reviewers using a double-blinded approach in the Rayyan web tool for reference management [15]. Before starting the selection process itself, the reviewers will participate in a training session to ensure a uniform understanding of the inclusion and exclusion criteria. An initial sample of 50 studies (titles and abstracts) will be reviewed by both reviewers for calibration, and any discrepancies will be discussed to refine the application of the criteria. A pre-designed form will be used to ensure consistency.

Initially, duplicates will be removed, followed by title and abstract screening, and subsequently by full-text reading of potentially relevant studies. With the support of the Rayyan platform (without the use of artificial intelligence functionalities), the removal of articles that do not meet the inclusion criteria will be recorded and reported in the scoping review. Discrepancies will be resolved by consensus or by a third reviewer. The selection process will follow the recommendations of PRISMA-ScR (PRISMA extension for Scoping Reviews) [16] and will be presented through a flowchart.

### Data extraction

Data will be extracted using a pre-designed form developed specifically for this review [17]. The form will include information about authorship, year of publication, country, objective, study summary, variables used to characterize the population, variables used to analyze the process of return to work after solid organ transplantation, evaluated outcomes, primary results, conclusions, and ethical considerations. These and other variables are available in Table 2.

**Table 2:** Data extraction form.

Data extraction	
**Article title**	
**First author**	
**Journal**	
**Year of publication**	
**Country in which the sample was collected**	
**Study type**	
**Study objective**	
**Methods**	
**Sample size (if applicable)**	
**Inclusion criteria**	
**Exclusion criteria**	
**Transplanted organ**	
**Living or deceased donor transplant?**	
**Sociodemographic variables — which and how categorized**	
**Psychosocial variables — which and how categorized**	
**Clinical variables — which and how categorized**	
**Organ-specific variables — which and how categorized**	
**Employment status — definition and categorization**	
**Pre-transplant work-related variables**	
**Post-transplant work-related variables**	
**Post-transplant changes in working conditions**	
**Mean time away from work before transplantation**	
**Mean time from post-transplant recovery to seeking work**	
**Mean time to return to work after transplantation**	
**Number of participants who returned to work**	
**Number of participants who did not return to work**	
**Reported predictors for return to work (or not) — if yes, which?**	
**Positive factors for return to work**	
**Negative factors for return to work**	
**Main conclusions**	
**Reported biases**	
**Ethical considerations**	
**Additional notes**	

Source: Authors, 2024.

Data extraction will be conducted independently by two reviewers using a pre-designed form. Before full extraction, the form will be piloted with a sample of 5 studies to ensure its suitability and make any necessary adjustments. Any discrepancies in the extraction process will be resolved through discussion between the reviewers, with the involvement of a third reviewer if needed. After extraction, a random sample comprising 20% of the studies will have their data verified by a third reviewer to ensure accuracy. Although formal quality assessment of studies is not typical in scoping reviews, notes on the methodological robustness of the studies will be included in the ‘Additional notes’ field of the extraction form.

### Data synthesis and presentation

The data synthesis will be performed in a narrative form, supported by tables, graphs, conceptual maps, and a world map to summarize findings and illustrate relationships [18]. A summary table will present the main characteristics of the included studies, highlighting authors, year, country, type of transplant, sample size, and key results. Relevant direct quotations will be incorporated to illustrate central themes.

Qualitative analyses, such as thematic analysis, may be performed to identify recurring themes in the included studies [19]. If conducted, this analysis will follow the six-phase approach proposed by Braun and Clarke, using NVivo software for coding and IRAMUTEQ for textual corpus analysis [20]. Quantitative analyses will include descriptive statistics (e.g., frequency counts, proportions, means, medians, and variability measures).

To enhance rigor and minimize potential bias, the results will be processed using appropriate software, subjected to statistical analyses, and peer evaluation will be conducted to identify potentially overlooked biases. The narrative synthesis will adhere to the Synthesis Without Meta-analysis (SWiM) guidelines [21], ensuring transparency and reproducibility.

Heterogeneity among studies will be narratively assessed, considering methodological, population, and contextual differences. Inconsistencies will be examined in light of study design, context, and sample characteristics. Literature gaps will be explicitly identified and discussed as potential areas for future research. The restriction to studies published in English and Portuguese will be acknowledged as a potential source of language bias.

### Consultation

Consultations with experts in the field of solid organ transplantation will be conducted to validate the findings and provide additional insights. A panel of 5–7 experts, including transplant surgeons, occupational rehabilitation specialists, and representatives of transplant patient associations, will be selected based on their experience and relevant publications. The experts will be recruited through email invitations, and their feedback will be integrated into the final synthesis of the results, contributing to the interpretation and contextualization of the findings.

The consultation process will be conducted after the initial data synthesis, comprising individual semi-structured interviews and a subsequent online focus group session to validate findings and refine interpretations. The interviews and group sessions will be recorded, transcribed, and thematically analyzed. The feedback from the experts will be documented in a summary table, indicating how each suggestion was incorporated into the final synthesis or, if not incorporated, the justification for such a decision.

This scoping review will strictly follow the PRISMA-ScR (PRISMA extension for Scoping Reviews) guidelines to ensure transparent and comprehensive reporting [16]. The PRISMA-ScR checklist will be used as a guide throughout the review process and the preparation of the final report. All 22 items of the checklist will be addressed, with particular emphasis on elements related to the review rationale, search strategy, study selection process, data extraction, and result synthesis. The completed PRISMA-ScR checklist will be included as an appendix in the final report, allowing readers to easily assess the review’s compliance with these guidelines. No significant adaptations to the PRISMA-ScR guidelines were made for this review, thus ensuring full adherence to the established standard. The completed checklist is available in S2 PRISMA-ScR-Checklist.

### Study timeline

The scoping review is proceeding according to the following schedule, ensuring the prospective nature of the review steps:

1. Protocol Development and Registration (completed: October 2023).
2. Search Strategy Refinement (completed: February 2025):
  - The search strategy was finalized in collaboration with a health sciences librarian.
3. Initial Database Searching (anticipated to commence after protocol publication, est. February 2026):
  - Systematic searches will be conducted across multiple databases.
4. Title and Abstract Screening (anticipated: March 2026):
  - Two independent reviewers will assess titles and abstracts using the Rayyan platform.
5. Full-Text Review and Data Extraction (anticipated: April-May 2026):
  - Full-text articles meeting the inclusion criteria will be retrieved and reviewed.
  - Data extraction will be carried out using a standardized pre-developed form.
6. Data Synthesis and Analysis (anticipated: June-July 2026):
  - Thematic analysis and data synthesis will be performed.
7. Manuscript Preparation and Submission (anticipated: August-September 2026).

The anticipated results, expected by September 2026, may include a comprehensive mapping of the literature on return to work after solid organ transplantation, identification of gaps in existing knowledge, and recommendations for future research and instrument development.

## Supporting information

S1 Table Search Strategy. (DOCX)

S2 Checklist PRISMA-ScR-Checklist. (DOCX)
