# Supplementary material for: Return to work outcomes in solid organ transplant recipients: A protocol for a global scoping review
Source: PLoS One. 2026 Apr 8;21(4):e0319873. doi: 10.1371/journal.pone.0319873 (PMC13061238; doi:10.1371/journal.pone.0319873)
Supplement: S1 Table — (DOCX) [file pone.0319873.s001.docx]

**S1 Table. Search Strategy.**

**Final search strategy across multiple databases and the count (n) of references retrieved from each database.**

| **Databases** | **Search strategy** | **Results (*n*)** |
| --- | --- | --- |
| PubMed | ("Return to Work"[Title/Abstract] OR "Work, Return to"[Title/Abstract] OR "Back-to-Work"[Title/Abstract] OR "Return-to-Work"[Title/Abstract] OR "Back to Work"[Title/Abstract] OR "Work, Back to"[Title/Abstract] OR Employment[Title/Abstract] OR "Employment Termination"[Title/Abstract] OR "Termination, Employment"[Title/Abstract] OR "Labor Force"[Title/Abstract] OR "Labor Forces"[Title/Abstract] OR "Precarious Employment"[Title/Abstract] OR "Employment, Precarious"[Title/Abstract] OR "Marginal Employment"[Title/Abstract] OR "Employment, Marginal"[Title/Abstract] OR "Employment Insecurity"[Title/Abstract] OR "Employment Insecurities"[Title/Abstract] OR "Insecurity, Employment"[Title/Abstract] OR "Employment Status"[Title/Abstract] OR "Status, Employment"[Title/Abstract] OR "Status, Occupational"[Title/Abstract] OR "Occupational Status"[Title/Abstract] OR Underemployment[Title/Abstract]) AND Transplantations NOT (Cell OR Cells) | 698 |
| SCOPUS | TITLE-ABSTRACT(({Return to Work} OR {Work, Return to} OR {Back-to-Work} OR {Return-to-Work} OR {Back to Work} OR {Work, Back to} OR Employment OR {Employment Termination} OR {Termination, Employment} OR {Labor Force} OR {Labor Forces} OR {Precarious Employment} OR {Employment, Precarious} OR {Marginal Employment} OR {Employment, Marginal} OR {Employment Insecurity} OR {Employment Insecurities} OR {Insecurity, Employment} OR {Employment Status} OR {Status, Employment} OR {Status, Occupational} OR {Occupational Status} OR Underemployment) AND Transplantations NOT (Cell OR Cells)) | 186 |
| EMBASE | (‘Return to Work’:ab,ti OR ‘Work, Return to’:ab,ti OR ‘Back-to-Work’:ab,ti OR ‘Return-to-Work’:ab,ti OR ‘Back to Work’:ab,ti OR ‘Work, Back to’:ab,ti OR Employment:ab,ti OR ‘Employment Termination’:ab,ti OR ‘Termination, Employment’:ab,ti OR ‘Labor Force’:ab,ti OR ‘Labor Forces’:ab,ti OR ‘Precarious Employment’:ab,ti OR ‘Employment, Precarious’:ab,ti OR ‘Marginal Employment’:ab,ti OR ‘Employment, Marginal’:ab,ti OR ‘Employment Insecurity’:ab,ti OR ‘Employment Insecurities’:ab,ti OR ‘Insecurity, Employment’:ab,ti OR ‘Employment Status’:ab,ti OR ‘Status, Employment’:ab,ti OR ‘Status, Occupational’:ab,ti OR ‘Occupational Status’:ab,ti OR Underemployment:ab,ti) AND Transplantations NOT (Cell OR Cells) | 448 |
| LILACS | (ti:"Return to Work" OR ab:"Return to Work" OR ti:"Work, Return to" OR ab:"Work, Return to" OR ti:"Back-to-Work" OR ab:"Back-to-Work" OR ti:"Return-to-Work" OR ab:"Return-to-Work" OR ti:"Back to Work" OR ab:"Back to Work" OR ti:"Work, Back to" OR ab:"Work, Back to" OR ti:Employment OR ab:Employment OR ti:"Employment Termination" OR ab:"Employment Termination" OR ti:"Termination, Employment" OR ab:"Termination, Employment" OR ti:"Labor Force" OR ab:"Labor Force" OR ti:"Labor Forces" OR ab:"Labor Forces" OR ti:"Precarious Employment" OR ab:"Precarious Employment" OR ti:"Employment, Precarious" OR ab:"Employment, Precarious" OR ti:"Marginal Employment" OR ab:"Marginal Employment" OR ti:"Employment, Marginal" OR ab:"Employment, Marginal" OR ti:"Employment Insecurity" OR ab:"Employment Insecurity" OR ti:"Employment Insecurities" OR ab:"Employment Insecurities" OR ti:"Insecurity, Employment" OR ab:"Insecurity, Employment" OR ti:"Employment Status" OR ab:"Employment Status" OR ti:"Status, Employment" OR ab:"Status, Employment" OR ti:"Status, Occupational" OR ab:"Status, Occupational" OR ti:"Occupational Status" OR ab:"Occupational Status" OR ti:Underemployment OR ab:Underemployment) AND Transplantations NOT (Cell OR Cells) | 128 |
| Web Of Science | (TI=("Return to Work" OR "Work, Return to" OR "Back-to-Work" OR "Return-to-Work" OR "Back to Work" OR "Work, Back to" OR Employment OR "Employment Termination" OR "Termination, Employment" OR "Labor Force" OR "Labor Forces" OR "Precarious Employment" OR "Employment, Precarious" OR "Marginal Employment" OR "Employment, Marginal" OR "Employment Insecurity" OR "Employment Insecurities" OR "Insecurity, Employment" OR "Employment Status" OR "Status, Employment" OR "Status, Occupational" OR "Occupational Status" OR Underemployment) OR AB=("Return to Work" OR "Work, Return to" OR "Back-to-Work" OR "Return-to-Work" OR "Back to Work" OR "Work, Back to" OR Employment OR "Employment Termination" OR "Termination, Employment" OR "Labor Force" OR "Labor Forces" OR "Precarious Employment" OR "Employment, Precarious" OR "Marginal Employment" OR "Employment, Marginal" OR "Employment Insecurity" OR "Employment Insecurities" OR "Insecurity, Employment" OR "Employment Status" OR "Status, Employment" OR "Status, Occupational" OR "Occupational Status" OR Underemployment) AND Transplantations NOT (Cell OR Cells) | 576 |
