# Supplementary material for: Return to work outcomes in solid organ transplant recipients: A protocol for a global scoping review
Source: PLoS One. 2026 Apr 8;21(4):e0319873. doi: 10.1371/journal.pone.0319873 (PMC13061238; doi:10.1371/journal.pone.0319873)
Supplement: S2 Checklist — (DOCX) [file pone.0319873.s002.docx]

**Preferred Reporting Items for Systematic reviews and Meta-Analyses extension for Scoping Reviews (PRISMA-ScR) Checklist**

| **SECTION** | **ITEM** | **PRISMA-ScR CHECKLIST ITEM** | **REPORTED ON PAGE #** |
| --- | --- | --- | --- |
| **TITLE** | | | |
| Title | 1 | Identify the report as a scoping review. | Return to work outcomes in solid organ transplant recipients: a protocol for a global scoping review |
| **ABSTRACT** | | | |
| Structured summary | 2 | Provide a structured summary that includes (as applicable): background, objectives, eligibility criteria, sources of evidence, charting methods, results, and conclusions that relate to the review questions and objectives. | Mapping return-to-work literature may reveal gaps in conceptualization, instruments, analyses, and key determinants. Following Joanna Briggs Institute methodology, this protocol aims to identify knowledge gaps and map the processes and outcomes in the literature on return to work after liver, kidney, heart, and lung transplantation. Following the Population, Concept, and Context strategy, this review is guided by the research question: “What has the literature shown about return to work after solid organ transplantation?”. This protocol was created and recorded on the Open Science Framework under DOI 10.17605/OSF.IO/Q6HVT. Database selection and search strategy were determined by a librarian specializing in health sciences. The literature search will be conducted in PubMed, Scopus, EMBASE, LILACS, and Web of Science databases. Eligible studies include primary and secondary research on return to work after transplantation, published in English or Portuguese, with no time restrictions. Two reviewers will independently perform the selection and data extraction. The data will be extracted using a pre-designed form to collect key details about the studies' origin, context, purpose, content, population, and variables related to the return-to-work process. These data will be synthesized following Synthesis Without Meta Analysis guidelines and summarized narratively using tables, graphs, alongside thematic analysis. |
| **INTRODUCTION** | | | |
| Rationale | 3 | Describe the rationale for the review in the context of what is already known. Explain why the review questions/objectives lend themselves to a scoping review approach. | Examining the current state of return to work among solid organ transplant recipients (liver, kidney, heart, and lung) can provide insights into the biopsychosocial factors influencing reintegration into the labor market. Identifying the key determinants of employment post-transplantation allows for a better assessment of challenges, barriers, and potential interventions. The absence of standardized instruments limits cross-study comparisons, conceptual clarity, and knowledge about critical variables such as immunosuppressant effects, pre- and post-transplant health conditions, and socioeconomic influences. Mapping the literature on return to work after transplantation will highlight existing gaps, enabling the development of new methods, validated instruments, and policy recommendations to improve employment outcomes for transplant recipients. |
| Objectives | 4 | Provide an explicit statement of the questions and objectives being addressed with reference to their key elements (e.g., population or participants, concepts, and context) or other relevant key elements used to conceptualize the review questions and/or objectives. | To identify knowledge gaps and map biopsychosocial processes and outcomes in the literature on return to work after liver, kidney, heart, and lung transplantation, considering transnational contexts and diverse methodological approaches. |
| **METHODS** | | | |
| Protocol and registration | 5 | Indicate whether a review protocol exists; state if and where it can be accessed (e.g., a Web address); and if available, provide registration information, including the registration number. | This protocol was developed in advance of the review and formally registered on the Open Science Framework (OSF) [13], a platform dedicated to promoting transparency, reproducibility, and open scientific practices. The record is publicly accessible and can be consulted through the following DOI: 10.17605/OSF.IO/Q6HVT. |
| Eligibility criteria | 6 | Specify characteristics of the sources of evidence used as eligibility criteria (e.g., years considered, language, and publication status), and provide a rationale. | This review will include all retrieved full-text studies that centrally examine the return-to-work process after solid organ transplantation. Eligible studies encompass primary research (observational and experimental studies) and secondary research (systematic reviews and meta-analyses). Both qualitative, quantitative, and mixed-methods approaches will be considered, with no hierarchy among study types. There will be no restrictions on publication date or country of origin, but only studies published in English or Portuguese will be included. |
| Information sources* | 7 | Describe all information sources in the search (e.g., databases with dates of coverage and contact with authors to identify additional sources), as well as the date the most recent search was executed. | Electronic databases: National Library of Medicine (PubMed), Scopus (Elsevier), Excerpta Medica Database (EMBASE), Latin American and Caribbean Health Sciences Literature (LILACS), and on the Web of Science platform. |
| Search | 8 | Present the full electronic search strategy for at least 1 database, including any limits used, such that it could be repeated. | Search strategy used for PubMed:  ("Return to Work"[Title/Abstract] OR "Work, Return to"[Title/Abstract] OR "Back-to- Work"[Title/Abstract] OR "Return-to-Work"[Title/Abstract] OR "Back to Work"[Title/Abstract] OR "Work, Back to"[Title/Abstract] OR Employment[Title/Abstract] OR "Employment Termination"[Title/Abstract] OR "Termination, Employment"[Title/Abstract] OR "Labor Force"[Title/Abstract] OR "Labor Forces"[Title/Abstract] OR "Precarious Employment"[Title/Abstract] OR "Employment, Precarious"[Title/Abstract] OR "Marginal Employment"[Title/Abstract] OR "Employment, Marginal"[Title/Abstract] OR "Employment Insecurity"[Title/Abstract] OR "Employment Insecurities"[Title/Abstract] OR "Insecurity, Employment"[Title/Abstract] OR "Employment Status"[Title/Abstract] OR "Status, Employment"[Title/Abstract] OR "Status, Occupational"[Title/Abstract] OR "Occupational Status"[Title/Abstract] OR Underemployment[Title/Abstract]) AND Transplantations NOT (Cell OR Cells) |
| Selection of sources of evidence† | 9 | State the process for selecting sources of evidence (i.e., screening and eligibility) included in the scoping review. | The study selection process will be conducted independently by two reviewers using a double-blinded approach in the Rayyan web tool for reference management. Before starting the selection process itself, the reviewers will participate in a training session to ensure a uniform understanding of the inclusion and exclusion criteria. An initial sample of 50 studies (titles and abstracts) will be reviewed by both reviewers for calibration, and any discrepancies will be discussed to refine the application of the criteria. |
| Data charting process‡ | 10 | Describe the methods of charting data from the included sources of evidence (e.g., calibrated forms or forms that have been tested by the team before their use, and whether data charting was done independently or in duplicate) and any processes for obtaining and confirming data from investigators. | Data will be extracted using a pre-designed form developed specifically for this review. The form will include information about authorship, year of publication, country, objective, study summary, variables used to characterize the population, variables used to analyze the process of return to work after solid organ transplantation, evaluated outcomes, primary results, conclusions, and ethical considerations. Before full extraction, the form will be piloted with a sample of 5 studies to ensure its suitability and make any necessary adjustments. Any discrepancies in the extraction process will be resolved through discussion between the reviewers, with the involvement of a third reviewer if needed. After extraction, a random sample comprising 20% of the studies will have their data verified by a third reviewer to ensure accuracy. |
| Data items | 11 | List and define all variables for which data were sought and any assumptions and simplifications made. | Data will be extracted using a pre-designed form developed specifically for this review. The form will include information about authorship, year of publication, country, objective, study summary, variables used to characterize the population, variables used to analyze the process of return to work after solid organ transplantation, evaluated outcomes, primary results, conclusions, and ethical considerations. Other items: Sample size (if applicable)  Inclusion criteria  Exclusion criteria  Transplanted organ  Living or deceased donor transplant?  Sociodemographic variables — which and how categorized  Psychosocial variables — which and how categorized  Clinical variables — which and how categorized  Organ-specific variables — which and how categorized  Employment status — definition and categorization  Pre-transplant work-related variables  Post-transplant work-related variables  Post-transplant changes in working conditions  Mean time away from work before transplantation  Mean time from post-transplant recovery to seeking work  Mean time to return to work after transplantation  Number of participants who returned to work  Number of participants who did not return to work  Reported predictors for return to work (or not) — if yes, which?  Positive factors for return to work  Negative factors for return to work  Main conclusions  Reported biases  Ethical considerations  Additional notes |
| Critical appraisal of individual sources of evidence§ | 12 | If done, provide a rationale for conducting a critical appraisal of included sources of evidence; describe the methods used and how this information was used in any data synthesis (if appropriate). | Does not apply. |
| Synthesis of results | 13 | Describe the methods of handling and summarizing the data that were charted. | The data synthesis will be performed in a narrative form, supported by tables, graphs, conceptual maps, and a world map to summarize findings and illustrate relationships. A summary table will present the main characteristics of the included studies, highlighting authors, year, country, type of transplant, sample size, and key results. Qualitative analyses, such as thematic analysis, may be performed to identify recurring themes in the included studies. If conducted, this analysis will follow the six-phase approach proposed by Braun and Clarke, using NVivo software for coding and IRAMUTEQ for textual corpus analysis. Quantitative analyses will include descriptive statistics (e.g., frequency counts, proportions, means, medians, and variability measures). The narrative synthesis will adhere to the Synthesis Without Meta-analysis (SWiM) guidelines. Heterogeneity among studies will be narratively assessed, considering methodological, population, and contextual differences. |
| **RESULTS** | | | |
| Selection of sources of evidence | 14 | Give numbers of sources of evidence screened, assessed for eligibility, and included in the review, with reasons for exclusions at each stage, ideally using a flow diagram. | Not applicable (protocol). |
| Characteristics of sources of evidence | 15 | For each source of evidence, present characteristics for which data were charted and provide the citations. | Not applicable (protocol). |
| Critical appraisal within sources of evidence | 16 | If done, present data on critical appraisal of included sources of evidence (see item 12). | Does not apply. |
| Results of individual sources of evidence | 17 | For each included source of evidence, present the relevant data that were charted that relate to the review questions and objectives. | Not applicable (protocol). |
| Synthesis of results | 18 | Summarize and/or present the charting results as they relate to the review questions and objectives. | Not applicable (protocol). |
| **DISCUSSION** | | | |
| Summary of evidence | 19 | Summarize the main results (including an overview of concepts, themes, and types of evidence available), link to the review questions and objectives, and consider the relevance to key groups. | Not applicable (protocol). |
| Limitations | 20 | Discuss the limitations of the scoping review process. | Not applicable (protocol). |
| Conclusions | 21 | Provide a general interpretation of the results with respect to the review questions and objectives, as well as potential implications and/or next steps. | Not applicable (protocol). |
| **FUNDING** | | | |
| Funding | 22 | Describe sources of funding for the included sources of evidence, as well as sources of funding for the scoping review. Describe the role of the funders of the scoping review. | This study received financial support from the National Council for Scientific and Technological Development (CNPq), Brazil, through a PhD scholarship awarded to VAP (grant number: 141532/2023-8). The funding does not cover other aspects of the research. The funder had no role in study design, data collection, analysis, interpretation, or manuscript preparation. The authors retained full independence in conducting the study and reporting the results. |

JBI = Joanna Briggs Institute; PRISMA-ScR = Preferred Reporting Items for Systematic reviews and Meta-Analyses extension for Scoping Reviews.

* Where *sources of evidence* (see second footnote) are compiled from, such as bibliographic databases, social media platforms, and Web sites.

† A more inclusive/heterogeneous term used to account for the different types of evidence or data sources (e.g., quantitative and/or qualitative research, expert opinion, and policy documents) that may be eligible in a scoping review as opposed to only studies. This is not to be confused with *information sources* (see first footnote).

‡ The frameworks by Arksey and O’Malley (6) and Levac and colleagues (7) and the JBI guidance (4, 5) refer to the process of data extraction in a scoping review as data charting*.*

§ The process of systematically examining research evidence to assess its validity, results, and relevance before using it to inform a decision. This term is used for items 12 and 19 instead of "risk of bias" (which is more applicable to systematic reviews of interventions) to include and acknowledge the various sources of evidence that may be used in a scoping review (e.g., quantitative and/or qualitative research, expert opinion, and policy document).

*From:* Tricco AC, Lillie E, Zarin W, O'Brien KK, Colquhoun H, Levac D, et al. PRISMA Extension for Scoping Reviews (PRISMAScR): Checklist and Explanation. Ann Intern Med. 2018;169:467–473. [doi: 10.7326/M18-0850](http://annals.org/aim/fullarticle/2700389/prisma-extension-scoping-reviews-prisma-scr-checklist-explanation).
